# Untargeted LC–MS/MS analysis reveals metabolomics feature of osteosarcoma stem cell response to methotrexate

**DOI:** 10.1186/s12935-020-01356-y

**Published:** 2020-06-24

**Authors:** Feng Wang, Zhiyu Zhang, Qin Li, Tao Yu, Chengbin Ma

**Affiliations:** 1 Department of Orthopedics, the Fourth Affiliated Hospital of China Medical University, Chongshan Road, Shenyang, 110032 Liaoning People’s Republic of China; 2 Center for Translational Medicine, the Fourth Affiliated Hospital of China Medical University, Chongshan Road, Shenyang, 110032 Liaoning People’s Republic of China

**Keywords:** Osteosarcoma (OS), Cancer stem cell (CSC), Chemo-resistance, Metabolomics, Methotrexate (MTX)

## Abstract

**Background:**

Cancer stem cell (CSC) is identified in osteosarcoma (OS) and considered resistant to chemotherapeutic agents. However, the mechanism of osteosarcoma stem cell (OSC) resistant to chemotherapy remains debatable and vague, and the metabolomics feature of OSC is not clarified.

**Materials and methods:**

OSC was isolated by using sphere forming assay and identified. Untargeted LC–MS/MS analysis was performed to reveal the metabolomics feature of OSC and underlying mechanisms of OSC resistant to methotrexate (MTX).

**Results:**

OSC was efficiently isolated and identified from human OS 143B and MG63 cell lines with enhanced chemo-resistance to MTX. The untargeted LC–MS analysis revealed that OSC showed differential metabolites and perturbed signaling pathways, mainly involved in metabolisms of fatty acid, amino acid, carbohydrate metabolism and nucleic acid. After treated with MTX, metabolomics feature of OSC was mainly involved metabolisms of amino acid, fatty acid, energy and nucleic acid. Moreover, compared with their parental OS cells response to MTX, the differential metabolites and perturbed signaling pathways were mainly involved in metabolism of amino acid, fatty acid and nucleic acid. What’s more, Rap1 signaling pathway and Ras signaling pathway were involved in OS cells and their SCs response to MTX.

**Conclusion:**

Sphere-forming assay was able to efficiently isolate OSC from human OS cell lines and the untargeted LC–MS/MS analysis was suggested a sufficient methodology to investigate metabolomics features of OS cells and OSCs. Moreover, the metabolomics features of OSCs response to MTX might reveal a further understanding of chemotherapeutic resistance in OS.

## Background

Cancer stem cell (CSC) is a small subpopulation of cancer cells that drives tumor growth and metastasis and is resistant to treatment. Evidence has confirmed that CSC can be isolated from various types of tumors [1–5]. In osteosarcoma (OS), CSC is first isolated and identified by Gibbs and colleagues [6, 7]. Thereafter, numerous studies are performed to explore the underlying mechanisms of CSC in OS development, progression and treatment. Chemotherapy is one of the most pivotal methods in treating OS and OS stem cell (OSC) is considered to be resistant to chemotherapeutic agents [8–10]. Wang et al. demonstrate that cancer stem cell-like side population (SP) cells shows high resistance against chemotherapeutic drugs and apoptosis [11]. In addition, OSCs are significant cisplatin-resistant compared with the non-CSCs [8, 9]. Results of one latest study shows that primary OS cells are resistant to methotrexate (MTX) treatment and MTX does not alter Sox-2 and OCT-4 expression, which are used to identify OSC from OS [12], suggesting OSC is resistant to MTX. Studies have been performed to explore the underlying mechanism of OSC resistant to chemotherapy. It is suggested that overexpression of TSSC3, telomerase and DNA repairment may be involved in drug resistance of OSC [13]. However, the underlying mechanism of OSC resistant to chemotherapy remains debatable and vague.

Reprogramming of metabolism is considered one of hallmarks of cancer and numerous studies are carried out to explore the signature and underlying mechanisms of cancer metabolisms [14–17]. A recent study reports that pancreatic CSCs have higher levels of glycolysis and increased de novo lipogenesis activity, but reduced mitochondrial OXPHOS levels, compared to bulk parental cancer cells [18]. Metabolomics is one of the most powerful and popular methodologies in studying cancer and CSC metabolism [19–21], which has been applied to study the serum and urinary metabolism, invasion and metastasis in OS and the results show that specific metabolism of some important metabolites is involved in the development and progression of OS [22–24]. Moreover, a recent study suggests metabolomics a potential method to measure cellular responses to different drugs and reveals specific metabolic features in OS after administration of chemotherapeutic treatment [25]. However, no studies have been conducted to investigate the metabolic feature of OSC and their response to MTX.

In the present study, the untargeted metabolomics methodology was applied to initially screen for changes in the metabolic profile of OSC and investigate the metabolic response of OSC to MTX, as this could help improve understanding of cellular responses to MTX and provide new endpoint markers of effect.

## Materials and methods

### Isolation and identification of OSCs

OSCs were isolated according to previously described methodologies [7, 26, 27] and detailed procedures were described in Additional file 1. Briefly, sphere forming assay was used to isolate OSCs from human metastatic 143B and tumorigenic MG63 cell lines (kindly gifted by Professor Zhengdong Cai, First Hospital of Shanghai, Shanghai, China). In order to identify OSCs, several biomarkers were selected and evaluated as previously described [28, 29]. Amongst these selected biomarkers, CD133 shows the stemness of OSCs and capacity of tumorigenicity [29], CD117 shows the capacity of sphere formation, tumorigenicity and drug resistance [29], OCT-4 and Sox-2 shows the capacity of stemness, sphere formation, tumorigenicity and invasiveness, and ALDH1 shows the capacity of spereformation and drug resistance [29]. Standard procedure were carried out as previously described (Additional file 1.) [26, 28, 30–33]. Immunofluorescent staining was used for assessing the expression of CD133 and OCT-4 between parental OS cell lines and their SCs, qRT‑PCR for expression of CD117 and immunoprecipitation and Western blot analysis for expression of ALDH1 and SOX-2 proteins. Moreover, proliferation activity of parental OS cell lines and their SCs was assessed with Cell Counting Kit 8 (CCK-8; Transgen, Beijing) assay in vitro and orthotopic xenograft animal experiments in vivo. Migration and invasion ability were analyzed with Matrigel^®^ migration/invasion assay.

### Drug cytotoxicity assays

The chemo-sensitivity of both parental OS cells and their SCs to MTX (Teva Pharma) was analyzed by using CCK-8 assay. The cells were seeded at 5 × 10^3^/well into 96-well plate and cultured for 12 h in DMEM or RPMI 1640 growth medium containing 10% FBS (Gibco, Grand Island, NY, USA). Cells were then treated with MTX at different concentrations in 100 µL of basic DMEM/RPMI 1640 medium. The control wells also received equivalent volume of the media without MTX. After 24 h of incubation, 20 µL CCK-8 reagent was added to each well, and incubated for additional 4 h. Absorbance of each well was determined at 450 nm. The absorbance obtained were proportional to the number of viable cells. The experiments were repeated trice to confirm the reproducibility. Cytotoxicity was expressed as the percentage of cells surviving in relation to untreated cells. GraphPad Prism for Windows (version 5.00, GraphPad Software, San Diego, CA, USA) was employed to produce dose–response curves by performing nonlinear regression analysis of the cell viability data. The 50% inhibitory concentration (IC50) was determined by probit regression to evaluate the sensitivity of parental OS cells and their SCs to MTX. The mean IC50 values were calculated from measurements of independent experiments (n = 3).

### Sample processing and metabolite extraction

Before the metabolomics analysis, the sample and metabolites were prepared as previously described [34]. After isolation of OSCs, parental OS cells and their SCs were enriched in monolayers and when reached 10^6^/ml, cells were treated with MTX, which was adjusted to achieve a final drug concentration, corresponding to the IC50 value as previously determined. All of the samples were divided into 8 groups: parental 143B as group 1A,143B stem cell (143B-SC) as 1B, 143B treated with MTX at a IC50 concentration (143B-IC50) as 2A and 143B-SC treated with MTX at a IC 50 concentration (143B-SC-IC50) as 2B, as well as MG63 as 3A, MG63-SC as 3B, MG63-IC50 as 4A and MG63-SC-IC50 as 4B.

After 48 h incubation, the medium was removed and the cells were washed twice with 3 mL of phosphate-buffered saline (PBS) at 37 °C. The metabolites were extracted from cell residue with 1 mL precooled methanol/acetonitrile/water (v/v, 2:2:1) under sonication for 1 h in ice baths. The mixture was incubated at − 20 °C for 1 h followed by centrifugation at 14,000*g*, 4 °C for 20 min, and then transferred to the sampling vial for LC–MS analysis.

Additionally, quality control (QC) samples were prepared to ensure data quality for metabolic profiling, by pooling aliquots of all samples that were representative of the all samples under analysis, and used for data normalization. QC samples were prepared and analyzed with the same procedure as that for the experiment samples in each batch. Dried extracts were then dissolved in 50% acetonitrile. Each sample was filtered with a disposable 0.22 µm cellulose acetate and transferred into 2 mL HPLC vials and stored at − 80 °C until analysis.

### LC–MS/MS analysis and data processing

LC–MS/MS analysis was performed as previously described LC–MS method [18, 31] with minor optimization, using a UPLC-ESI-Q-TOF–MS system (UHPLC, 1290 Infinity LC, Agilent Technologies, Santa Clara, CA, USA) coupled TripleTOF 5600 (AB Sciex, Framingham, MA, USA). For hydrophilic interaction liquid chromatography (HILIC) separation, samples were analyzed using a 2.1 mm × 100 mm ACQUIY UPLC BEH 1.7 μm column (Waters, Ireland). The flow rate was 0.5 mL/min and the mobile phase contained: A = 25 mM ammonium acetate and 25 mM ammonium hydroxide in water, and B = acetonitrile (ACN). The gradient was 95% B for 0.5 min and was linearly reduced to 65% in 6.5 min, then reduced to 40% in 2 min. After maintained for 1 min, it was increased to 95% in 1.1 min, with 5 min re-equilibration period employed. Both electrospray ionization (ESI) positive-mode and negative-mode were applied for MS data acquisition. The ESI source conditions were set as follows: Ion Source Gas 1 as 60, Ion Source Gas 2 as 60, curtain gas as 30, source temperature: 600 °C and IonSpray Voltage Floating (ISVF) ± 5500 V. In MS only acquisition, the instrument was set to acquire over the m/z range 60–1200 Da, and the accumulation time for TOF MS scanning was set at 0.15 s/spectra. In auto MS/MS acquisition, the instrument was set to acquire over the m/z range 25–1200 Da. The accumulation time for product- ion scan was set at 0.03 s/spectra. The product-ion scan was acquired using information dependent acquisition with high sensitivity mode selected. The collisional energy was fixed at 30 V with ± 15 eV. Declustering potential was set as ± 60 V.

QC samples were prepared by pooling aliquots of all samples that were representative of the samples under analysis, and used for data normalization. Blank samples (75% ACN in water) and QC samples were injected every six samples during acquisition.

### Data preprocessing and filtering

The raw MS data were converted to MzXML files using ProteoWizard MSConvert and processed using XCMS for feature detection, retention time correction and alignment. The metabolites were identified by accuracy mass (< 25 ppm) and MS/MS data which were matched with a standards database. In the extracted-ion features, only the variables having more than 50% of the nonzero measurement values in at least one group were kept.

### Multivariate statistical analysis

SIMCAP software (Version 14.0, Umetrics, Umeå, Sweden) was used for all multivariate data analyses and modelings. Data were mean-centered using Pareto scaling. Models were built on principal component analysis (PCA), orthogonal partial least-square discriminant analysis (PLS-DA) and partial least-square discriminant analysis (OPLS-DA). All the models evaluated were tested for over fitting with methods of permutation tests. The descriptive performance of the models was determined by R^2^X (cumulative) (perfect model: R^2^X (cum) = 1) and R^2^Y (cumulative) (perfect model: R^2^Y (cum) = 1) values while their prediction performance was measured by Q2 (cumulative) (perfect model: Q2 (cum) = 1) and a permutation test (n = 200). The permuted model should not be able to predict classes: R2 and Q2 values at the Y-axis intercept must be lower than those of Q2 and the R2 of the non-permuted model. OPLS-DA allowed the determination of discriminating metabolites using the variable importance on projection (VIP). The VIP score value indicates the contribution of a variable to the discrimination between all the classes of samples. Mathematically, these scores are calculated for each variable as a weighted sum of squares of PLS weights. The mean VIP value is 1, and usually VIP values over 1 are considered as significant. A high score is in agreement with a strong discriminatory ability and thus constitutes a criterion for the selection of biomarkers.

The discriminating metabolites were obtained using a statistically significant threshold of VIP values obtained from the OPLS-DA model and two-tailed Student’s *t* test (*P* value) on the normalized raw data at univariate analysis level. The *P* value was calculated by one-way analysis of variance (ANOVA) for multiple groups analysis. Metabolites with VIP values greater than 1.0 and p value less than 0.05 were considered to be statistically significant metabolites. Fold change was calculated as the logarithm of the average mass response (area) ratio between two arbitrary classes. On the other side, the identified differential metabolites were used to perform cluster analyses with R package.

### KEGG enrichment analysis

To identify the perturbed biological pathways, KEGG ID Mapping was performed on the metabolites with significant difference among the comparison groups and submitted to KEGG website for related pathway analysis. The pathways with *P* < 0.05 was statistically of significant difference between groups. (http://www.kegg.jp). KEGG enrichment analyses were carried out with the Fisher’s exact test, and FDR correction for multiple testing was performed. Enriched KEGG pathways were nominally statistically significant at the *P* < 0.05 level.

### Statistical analysis

All the data were analyzed using SPSS 19.0 software. The data are shown as mean ± standard deviation (X ± SD). Cell proliferation is shown as optical density (OD) value. Cell migration and invasion are shown as cell amount. Student’s *t* test One-way ANOVA was used for comparisons among all treatments. Least significant difference (LSD) was used for multiple comparisons when there was homogeneity of variance, while Dunnett’s T3 test was used with heterogeneity of variance. *P *< 0.05 was considered to indicate a significant difference.

VIP was obtained from OPLS-DA model and used to screen the differential metabolites. In our study, VIP > 1 was used preliminarily to screen differential metabolites between groups. Differences were deemed significant for *P* values < 0.05. The metabolites with VIP > 1 and *P* < 0.05 were significantly differential metabolites between groups.

## Results

### Isolation and identification of OSCs

**Fig. 1:**
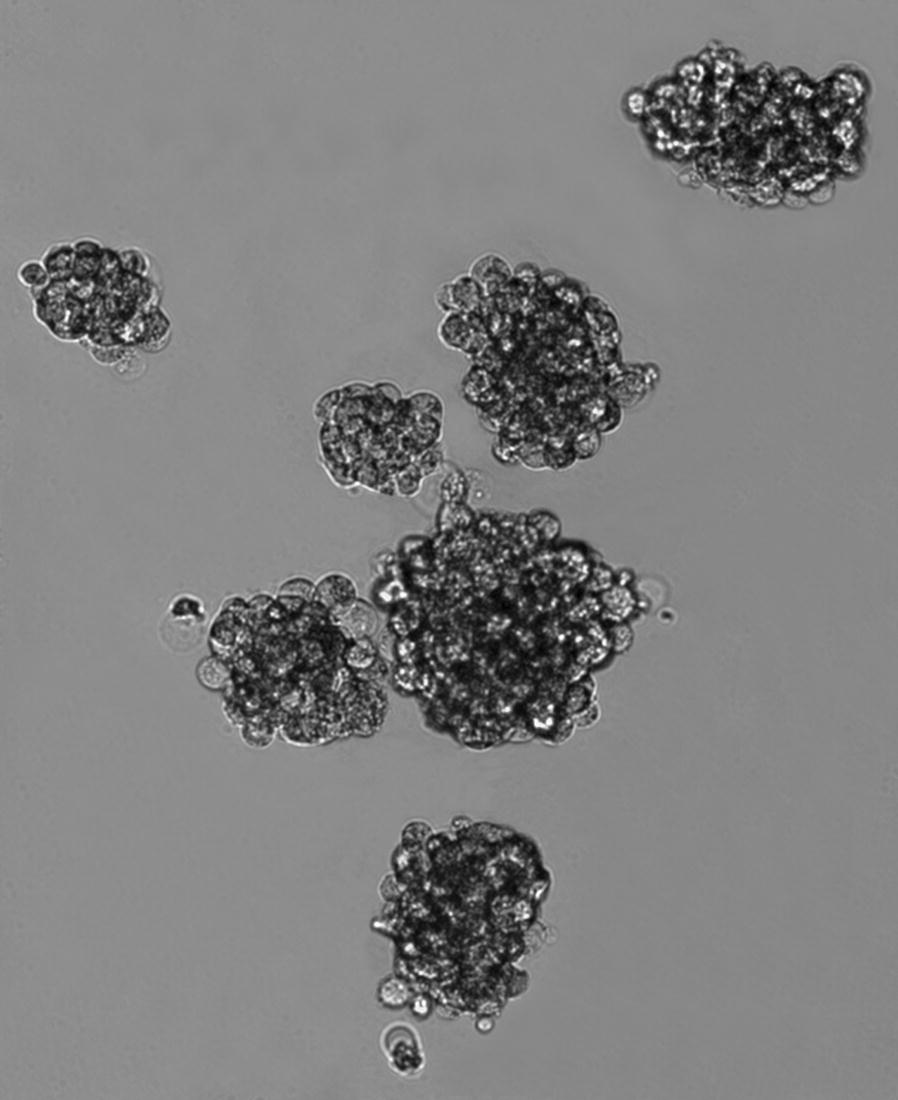
Spherical colonies (sarcospheres) generated from single-cell suspensions of OS cells cultured in serum-free medium supplemented with growth factors in non-adherent conditions. OS cells gradually aggregated and formed tumor spheres (Original magnification: 200 ×)

**Fig. 2:**
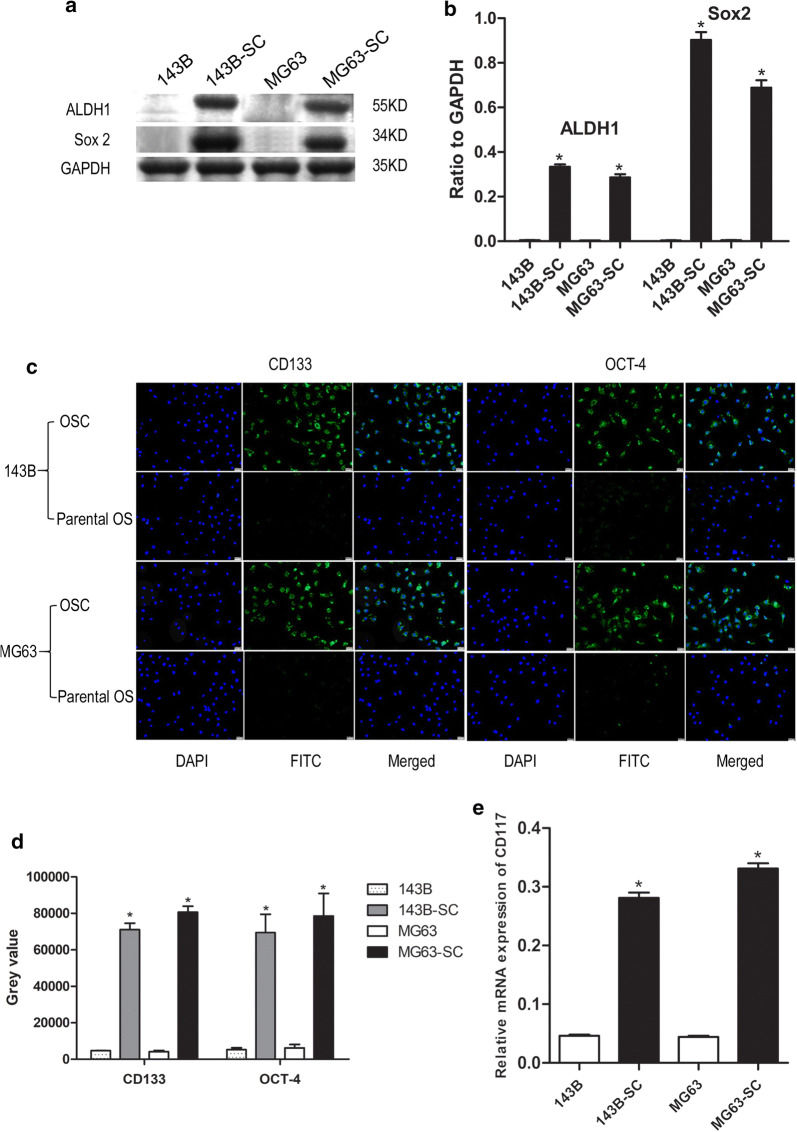
Measurement of the expression of targeted genes, ALDH1, Sox2, CD133, OCT-4 and CD 177 in OS and their SCs. **a** Immunoblotting assay of ALDH1, Sox-2 and GAPDH, **b** Statistical analysis of the expression of ALDH1 and Sox-2, **c** Immunofluorescence assay of CD133 and OCT-4 (Original magnification: 200 ×), **d** Statistical analysis of the expression of CD133 and Oct-4, **e** Statistical analysis of the expression of CD117 at transcriptional level by qRT-PCR. *compared with their parental osteosarcoma cells, *P *< 0.05. *SC* stem cell, *OS* osteosarcoma, *ALDH1* aldehyde dehydrogenase 1, *Sox-2* sex determining region Y (SRY)-like box 2, *OCT-4* octamer‑binding transcription factor 4, *FITC* fluorescein isothiocyanate, *DAPI* 4′,6-diamidino-2-phenylindole, *GAPDH* glyceraldehyde-3-phosphate dehydrogenase

The methodology of isolation and identification of OSCs were described in detail in the Supplementary file 1. Briefly, parental 143B and MG63 cell lines were used to isolate CSCs with sphere‑forming assay. After 7 days incubation, the OS cells gradually detached from the culture dishes, aggregated and became spheres-forming OSCs (Fig. 1). In order to characterize the stem-like properties of isolated OS cells, the expression of progenitor/stem cell genes Sox2 (Fig. 2a, b), CD133, OCT-4 (Fig. 2c, d) and CD 177 (Fig. 2e) were evaluated, respectively. An upregulated expression of Sox-2 was also observed to show a strong stemness in OSCs [13, 29]. Moreover, the majority of isolated OSCs showed elevated CD133, OCT-4 and CD 117 expression, which suggested strong tumorigenicity and self-renewal capacities for sphere forming cells [13, 29].

**Fig. 3:**
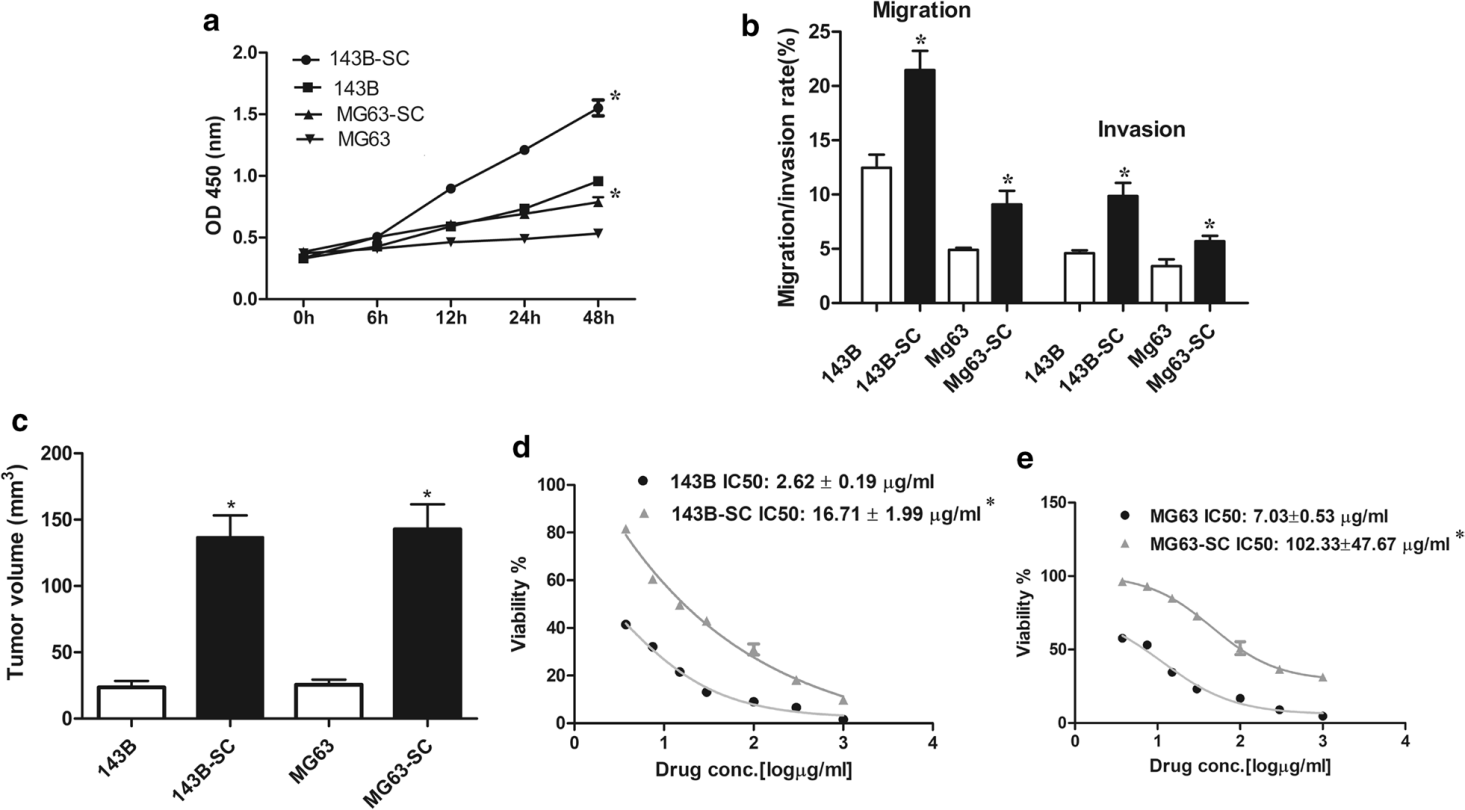
The measurement of tumorigenic potential in osteosarcoma (OS) and their stem cells. **a** Proliferation rate of OS and their stem cells was determined by CCK8 assay; **b** Migration and invasion rate was determined by Matrigel^®^ assay; **c** Orthotopic xenograft models were performed and at 3 weeks after injection, the tumor volume was measured; **d, e** Methotrexate cytotoxicity assays were performed by using CCK8 assay and IC50 was estimated with GraphPad Prism for Windows. *compared with their parental OS cells, *P *< 0.05. *SC* stem cell

What’s more, the enhanced tumorigenic potential was evaluated in vitro and in vivo. Briefly, a CCK-8 assay was performed to evaluate the proliferation rates of parental OS cells and their OSCs and the results showed that OSCs exhibited an increased proliferation capability compared with parental cells (Fig. 3a), while Matrigel^®^ migration/invasion assays showed that OSCs had increased migration and invasion capabilities (Fig. 3b, *P* < 0.05). To further validate the enhanced tumorigenicity of OSCs in vivo, xenograft tumorigenicity assay by using BALB/c nude mice was performed. After 3 weeks of xenotransplantation, the tumor volume was significantly increased in OSC‑transplanted mice (Fig. 3c; *P* < 0.05). What’s more, the sensitivity to the treatment of MTX was evaluated. Notably, OSCs isolated from 143B and MG63 cell lines were more resistant to MTX (Fig. 3d, e, respectively) in dose-dependent concentrations compared with parental OS cells (*P* < 0.05) and an increased IC50 was observed for OSCs. In addition, ALDH1 showed an increased expression level in OSCs (Fig. 2a, b), which was suggested to indicate enhanced chemotherapeutic resistance in OS [35].

### Quality evaluation of LC–MS/MS analysis

**Fig. 4:**
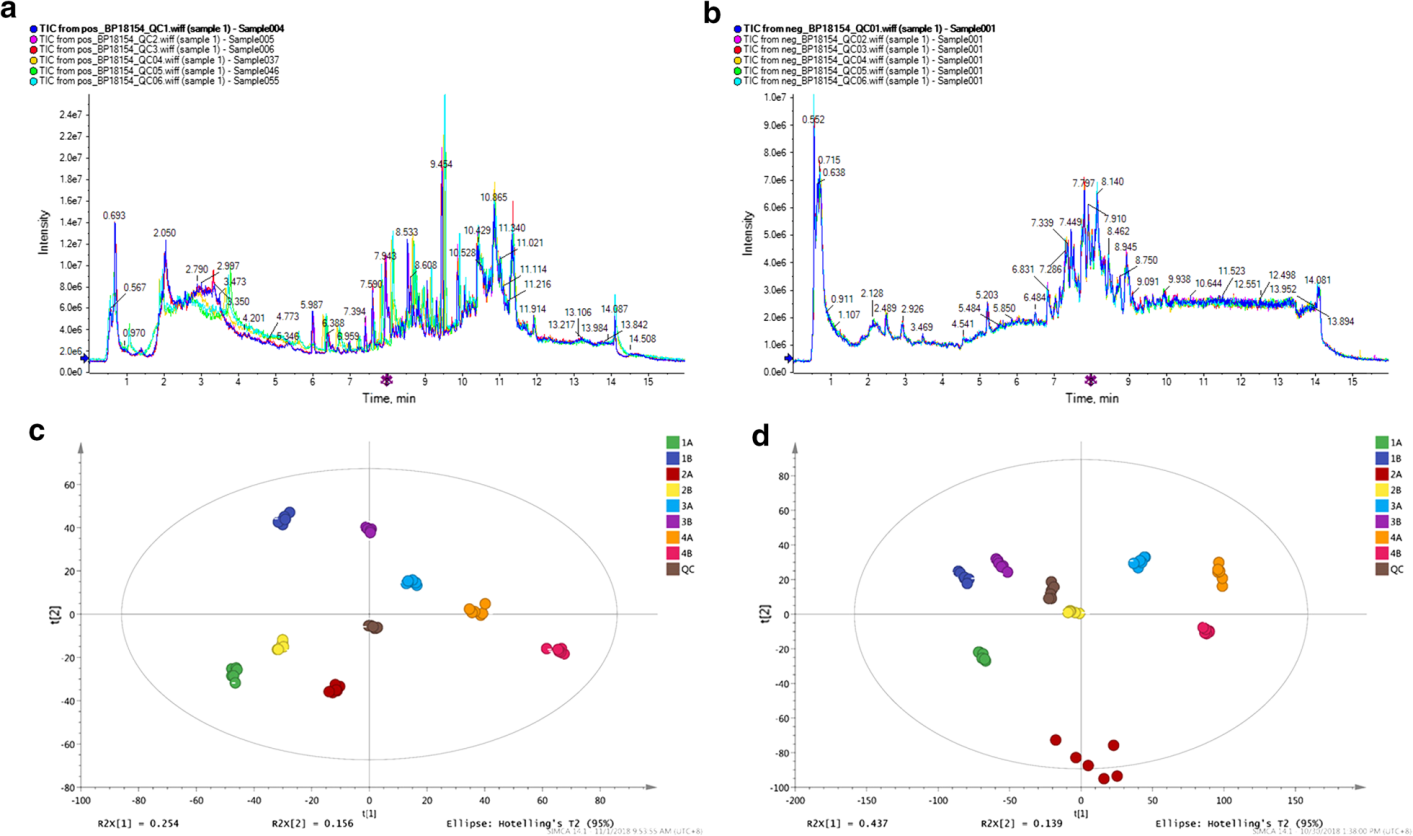
TIC analysis and PCA analysis of all samples. The results TIC analysis showed that the response intensity and retention time of each chromatographic peak under positive **a** and negative **b** ion detection modes were basically overlapped. PCA analysis showed that all samples of every group in positive **c** and negative **d** ion mode were closely clustered, indicating good repeatability in this study

Comparison of total ion current (TIC) of quality control (QC), PCA analysis and PLS-DA were performed to evaluate the reliability and stability of current metabolomics analysis. TIC of all samples under positive and negative ion detection modes were super-imposed and compared, as shown in Fig. 4a, b. The results showed that the response intensity and retention time of each chromatographic peak were basically overlapped, indicating that the variation caused by instrument errors was small and the data quality was reliable during the whole experiment. Moreover, the ion peaks of all the experimental samples and QC samples, extracted by XCMS software, were processed by Pareto-scaling for PCA analysis. PCA analysis showed that all samples of every group in positive and negative ion mode were closely clustered (Fig. 4c, d and Table 1.), which indicated good repeatability in this study. In addition, PLS-DA and OPLS-DA were performed to characterize metabolic patterns of all samples. Our results demonstrated that the metabolic profiles were not significantly different between two groups (Table 2).

**Table 1:** Summary of PCA evaluation

Title	Ion model	A	R^2^X (cum)	Q^2^ (cum)
QC	Positive	6	0.808	0.757
QC	Negative	8	0.803	0.7
1A vs. 1B	Positive	2	0.721	0.579
1A vs. 1B	Negative	2	0.734	0.57
2A vs. 2B	Positive	2	0.764	0.641
2A vs. 2B	Negative	2	0.693	0.495
3A vs. 3B	Positive	2	0.815	0.706
3A vs. 3B	Negative	2	0.643	0.423
4A vs. 4B	Positive	2	0.671	0.466
4A vs. 4B	Negative	2	0.721	0.54

*QC* quality control, *PCA* principal component analysis

**Table 2:** Summary of PLS-DA and OPLS-DA evaluation

Title	Ion model	PLS-DA				OPLS-DA			
		A	R^2^X (cum)	R^2^Y (cum)	Q^2^ (cum)	A	R^2^X (cum)	R^2^Y (cum)	Q^2^ (cum)
1A vs 1B	Positive	2	0.706	1	0.998	1 + 1+0	0.706	1	0.997
1A vs 1B	Negative	2	0.732	1	0.997	1 + 1+0	0.732	1	0.996
2A vs 2B	Positive	2	0.753	0.995	0.983	1 + 1+0	0.753	0.995	0.982
2A vs 2B	Negative	2	0.671	1	0.997	1 + 1+0	0.671	1	0.995
3A vs 3B	Positive	2	0.807	1	0.998	1 + 1+0	0.807	1	0.998
3A vs 3B	Negative	2	0.633	1	0.994	1 + 1+0	0.633	1	0.992
4A vs 4B	Positive	2	0.642	1	0.994	1 + 1+0	0.642	1	0.993
4A vs 4B	Negative	2	0.683	1	0.995	1 + 1+0	0.683	1	0.996

*PLS-DA* partial least squares to latent structure-discriminant analysis, *OPLS-DA* orthogonal partial last square-discriminant analysis

### Metabolomics feature of OSCs and their response to MTX

#### Differential metabolites and enriched pathways between parental OS cell and OSC

In 143B cell line, 94 differential metabolites were identified between parental OS cells and their stem cells, while 120 metabolites were identified between MG63 cells and their stem cells. In order to identify common differential metabolites between different cell lines, the identified metabolites were cross-compared and the results showed that a total of 57 common differential metabolites were finally identified between parental 143B and MG63 cells and their OSCs. In order to gain a better understanding of metabolomics feature of OSC, further screening was performed to assess the expression consistency of the metabolites between 143B and MG63 cell lines and their OSCs. The expression of 37 differential metabolites were uniform, with 14 upregulated (FC < 1) and 23 downregulated (FC > 1). 20 metabolites were involved in fatty acid metabolism, 10 in amino acid metabolism, 2 in carbohydrate metabolism and 5 in nucleic acid metabolism (Table 3).

**Table 3:** Summary of differential metabolites between OS and OSC

	Metabolite	143B			MG63		
		VIP	*P* value	FC	VIP	*P* value	FC
Amino acid	Prolyl-Threonine	2.59	6.43 × 10^−15^	0.04	1.76	1.72 × 10^−12^	0.13
	S-Methyl-5′-thioadenosine	2.23	3.60 × 10^−05^	1.21	3.38	5.45 × 10^−12^	2.44
	S-Adenosyl-L-homocysteine	3.54	1.03 × 10^−08^	35.32	1.00	1.95 × 10^−05^	11.05
	NAAG	1.06	5.40 × 10^−07^	1.61	1.42	1.92 × 10^−08^	7.63
	Glutathione	12.13	1.11 × 10^−14^	16.90	5.25	2.69 × 10^−08^	10.60
	Glutathione disulfide	1.04	1.21 × 10^−4^	1.13	1.70	1.07 × 10^−05^	1.80
	L-Malic acid	2.71	7.98 × 10^−10^	8.12	1.73	3.57 × 10^−09^	1.53
	N-Acetyl-l-aspartic acid	3.86	7.27 × 10^−11^	3.10	4.39	3.57 × 10^−08^	1.53
	Harman	7.24	7.07 × 10^−09^	0.66	7.57	1.08 × 10^−11^	0.39
	Indole-3-carboxylic acid	1.56	7.89 × 10^−09^	29.18	1.08	2.07 × 10^−10^	15.99
Fatty acids	Erucic acid	1.25	4.48 × 10^−05^	0.76	2.02	5.28 × 10^−11^	0.18
	Erucamide	11.38	1.41 × 10^−05^	0.71	16.66	6.28 × 10^−10^	0.14
	Linoleic acid	3.90	2.82 × 10^−10^	7.63	2.53	4.03 × 10^−4^	4.64
	Stearic acid	3.78	1.54 × 10^−07^	0.63	3.89	2.15 × 10^−10^	0.48
	Capric acid	2.57	4.40 × 10^−09^	0.62	1.77	2.32 × 10^−08^	0.66
	Desmosterol	3.12	5.16 × 10^−09^	0.23	3.68	8.14 × 10^−11^	0.25
	Undecanoic Acid	1.96	2.63 × 10^−10^	0.41	1.17	5.61 × 10^−10^	0.49
	Stearoylcarnitine	6.89	2.06 × 10^−12^	0.19	9.86	2.68 × 10^−11^	0.33
	LysoPE(16:0/0:0)	3.56	3.47 × 10^−10^	2.29	4.65	3.28 × 10^−12^	4.89
	25-Hydroxycholesterol	3.23	1.12 × 10^−08^	0.21	2.24	1.51 × 10^−09^	0.15
	LysoPE(18:0/0:0)	1.73	6.86 × 10^−11^	2.91	1.44	5.23 × 10^−11^	2.48
	LysoPC(16:0)	13.45	2.66 × 10^−10^	1.80	14.17	1.02 × 10^−12^	3.12
	LysoPC(O-18:0)	5.87	3.91 × 10^−12^	3.21	2.07	1.50 × 10^−07^	1.23
	SOPC	3.60	6.80 × 10^−3^	0.63	4.52	2.21 × 10^−13^	0.28
	Phosphorylcholine	1.58	1.63 × 10^−09^	0.25	2.97	2.93 × 10^−13^	0.07
	16-Hydroxypalmitic acid	1.23	5.22 × 10^−09^	3.13	2.60	2.63 × 10^−14^	4.86
	Oleic acid	1.53	4.42 × 10^−05^	1.72	2.49	4.40 × 10^−07^	2.26
	LysoPE(16:0/0:0)	4.26	1.74 × 10^−10^	2.33	8.06	1.48 × 10^−12^	3.97
	Retinol	2.72	1.05 × 10^−14^	10.70	1.83	1.85 × 10^−12^	8.09
	Geranyl diphosphate	3.51	6.76 × 10^−13^	0.03	4.06	3.83 × 10^−16^	0.02
Carbohydrates	UDP-d-Glucose	3.23	7.36 × 10^−09^	4.24	2.32	7.16 × 10^−05^	2.24
	d-Fructose 1,6-bisphosphate	3.38	1.98 × 10^−06^	0.13	1.35	1.08 × 10^−07^	0.49
Nucleic acid	2′-O-methylcytidine	3.92	1.37 × 10^−11^	0.26	4.05	4.73 × 10^−14^	0.11
	Adenosine 3′-monophosphate	4.67	9.90 × 10^−16^	13.53	2.04	8.97 × 10^−10^	7.84
	UDP	3.86	9.19 × 10^−10^	6.38	3.11	1.14 × 10^−09^	2.26
	UTP	2.33	7.48 × 10^−05^	5.58	2.10	1.42 × 10^−3^	3.84
	dGTP	9.72	1.21 × 10^−05^	2.47	5.47	1.90 × 10^−2^	1.32

*VIP* Variable Importance for the Project, *FC* fold change, *NAAG* N-Acetylaspartylglutamate, *SOPC* 1-Stearoyl-2-oleoyl-sn-glycerol 3-phosphocholine, *UDP-d-Glucose* Uridine diphosphate glucose, *UDP* Uridine 5′-diphosphate, *UTP* Uridine 5′-triphosphate, *dGTP* deoxyguanosine triphosphate

**Fig. 5:**
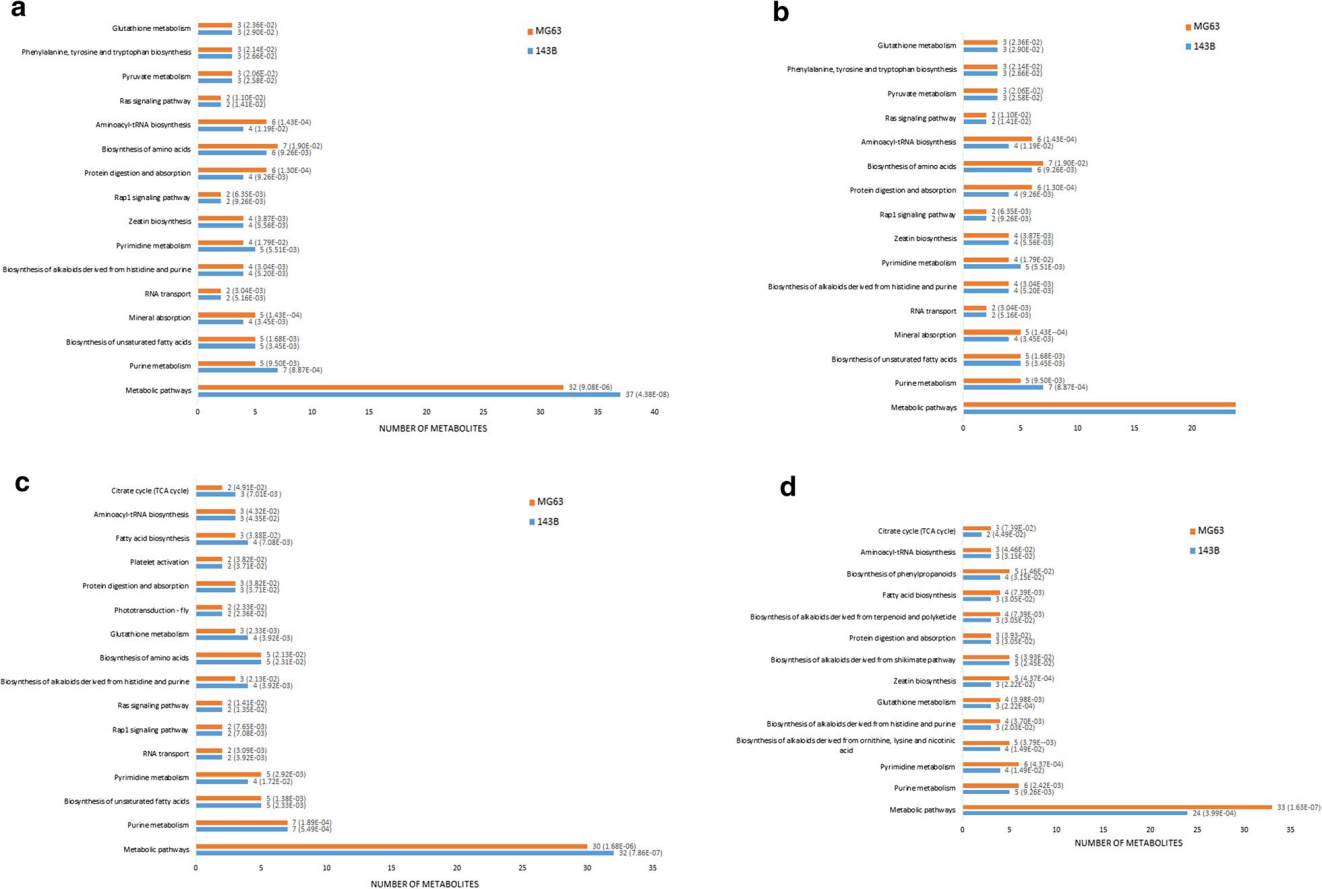
Perturbed biological signaling pathways were identified by using KEGG pathway analysis. **a** Perturbed biological pathways between OS and OSC, **b** Perturbed biological pathways between OS cells and OS cells after administration of MTX at IC50, **c** Perturbed biological pathways between OSCs and OSCs after administration of MTX at IC50, **d** Perturbed biological pathways between OS cells response to MTX and OSCs response to MTX at IC50, respectively. N: differentially expressed metabolites involved in the pathway. Numbers cited in scientific notation in the bracket: statistical *P* value

On the basis of KEGG PATHWAY Database (http://www.genome.jp/kegg/), perturbed biological pathways between OS cells and their stem cells were identified. In 143B cell line, 31 pathways were identified between parental OS cells and their stem cells, while there were 27 pathways identified between MG63 cells and their stem cells. Common perturbed biological pathways between cell lines were further screened by cross-comparing the results. In total, there were 17 pathways in common were finally identified between parental 143B and MG63 cells and their OSCs (Fig. 5a). Most of the identified pathways were involved in fatty acid, amino acid and nucleic acid metabolism. Some pathway (Biosynthesis of phenylpropanoids) [36] was involved in protecting stem cell under oxidative stress.

#### Differential metabolites and enriched pathways of OSC response to MTX

One of the most important characteristics for OSCs is their increased resistance to chemotherapeutic agents [14]. After isolation of OSCs with sarcosphere-forming assay, MTX was administered to parental OS cells and their SCs at an IC50 concentration. After 48 h, the differential metabolites and pathways were identified. In order to reveal the metabolomics feature of OSC responding to MTX, we initially analyzed the metabolomics feature of OS cells responding to MTX and the results showed that the differential metabolites and perturbed biological pathways were mainly involved in amino acid, fatty acid and nucleic acid metabolisms and energy metabolism (Table 4 and Fig. 5b). While metabolomics results of OSC responding to MTX showed that the differential metabolites were mainly involved amino acid metabolism (n = 28), fatty acid metabolism (n = 24), other 3 were involved in energy and nucleic acid metabolism (Table 5). KEGG pathway analysis verified the results of metabolites (Fig. 5c). In order to better understand the metabolic feature of OSC responding to MTX, the differential metabolites of OSC responding to MTX at an IC50 concentration were identified. Compared with metabolites of their parental OS cells responding to MTX, 23 metabolites were differential, which involved in amino acid metabolism, fatty acid metabolism and nucleic acid metabolism (Table 6). Fourteen pathways were identified, including citrate cycle (TCA cycle) pathway, which verified the results of the differential metabolites (Fig. 5d). Moreover, KEGG pathway analysis revealed that Rap1 signaling pathway and Ras signaling pathway were involved in parental OS cell and their SCs responding to MTX at an IC50 concentration, which were suggested in various cancer cell biological functions, including cell migration, invasion, proliferation and gene expression and activation [37, 38].

**Table 4:** Summary of differential metabolites between OS and OS-IC50

	Metabolite	143B			MG63		
		VIP	*P* value	FC	VIP	*P* value	FC
Amino acids	Lysyl-Glycine	1.83	1.56 × 10^−04^	0.14	2.37	4.07 × 10^−04^	0.67
	Prolyl-Valine	1.72	1.35 × 10^−07^	0.13	1.88	1.85 × 10^−05^	0.67
	Alanyl-Isoleucine	1.48	1.03 × 10^−09^	0.05	1.25	1.42 × 10^−05^	0.83
	l-Proline	1.11	3.07 × 10^−04^	0.09	2.17	5.75 × 10^−04^	0.36
	L-prolyl-l-phenylalanine	1.58	1.73 × 10^−04^	0.25	1.85	1.52 × 10^−03^	0.82
	Histidinyl-Methionine	1.53	1.37 × 10^−07^	0.16	4.03	6.46 × 10^−07^	0.17
	Phenylalanyl-Alanine	5.82	5.70 × 10^−09^	0.04	8.22	2.12 × 10^−08^	0.68
	Isoleucyl-Tyrosine	2.46	1.04 × 10^−05^	0.12	3.29	3.24 × 10^−06^	0.59
	Phenylalanyl-Glutamate	1.68	8.19 × 10^−07^	0.14	2.07	6.45 × 10^−06^	0.59
	Ornithine	1.10	8.96 × 10^−06^	0.05	1.51	1.09 × 10^−09^	0.48
	Valyl-Tryptophan	1.77	7.45 × 10^−10^	0.09	3.47	2.38 × 10^−09^	0.37
	NAAG	1.27	2.53 × 10^−10^	6.58	1.37	1.11 × 10^−04^	2.12
	Alpha-N-Phenylacetyl-l-glutamine	1.59	7.87 × 10^−08^	0.03	2.01	5.01 × 10^−06^	0.43
	N-(omega)-Hydroxyarginine	1.03	4.98 × 10^−04^	0.21	1.15	2.16 × 10^−03^	0.44
	gamma-L-Glutamyl-l-phenylalanine	1.22	1.12 × 10^−08^	0.18	1.13	3.80 × 10^−07^	0.62
	S-Adenosyl-L-homocysteine	2.33	1.61 × 10^−06^	3.20	1.22	4.99 × 10^−05^	6.00
	Phenylalanyl-Tryptophan	1.35	5.22 × 10^−08^	0.14	1.51	1.87 × 10^−06^	0.67
	Valyl-Methionine	1.33	4.65 × 10^−08^	0.10	1.37	2.91 × 10^−03^	0.61
	Leucyl-Glutamine	1.56	8.20 × 10^−08^	0.05	2.15	3.86 × 10^−08^	0.24
	L-Malic acid	2.49	1.79 × 10^−08^	3.36	1.56	3.83 × 10^−08^	1.38
	Citrate	4.62	1.53 × 10^−10^	3.11	2.12	1.07 × 10^−06^	1.37
	Glutathione	5.65	2.45 × 10^−06^	1.51	5.55	1.29 × 10^−06^	2.62
	Tyrosyl-Serine	1.26	7.82 × 10^−06^	0.10	1.72	6.27 × 10^−08^	0.53
	Methionyl-Tyrosine	1.22	2.91 × 10^−07^	0.10	1.33	2.37 × 10^−06^	0.60
	Phenylalanylphenylalanine	2.84	7.23 × 10^−09^	0.09	3.66	3.80 × 10^−08^	0.64
	Isoleucyl-Tryptophan	1.85	7.53 × 10^−06^	0.14	3.56	1.89 × 10^−05^	0.42
	Phenylalanyl-Tyrosine	1.94	7.19 × 10^−06^	0.21	1.96	2.50 × 10^−02^	0.65
	7,8-Dihydrobiopterin	1.41	2.99 × 10^−06^	0.05	2.74	1.58 × 10^−08^	0.41
Fatty acids	Desmosterol	2.62	1.23 × 10^−06^	0.21	1.25	1.99 × 10^−03^	0.79
	7-Ketocholesterol	1.18	1.61 × 10^−03^	1.72	1.33	1.87 × 10^−05^	2.53
	Undecanedioic acid	1.06	4.26 × 10^−05^	0.27	1.18	5.06 × 10^−05^	0.69
	Erucamide	11.91	1.43 × 10^−06^	2.93	4.20	1.68 × 10^−06^	1.31
	Linoleic acid	1.47	2.22 × 10^−02^	1.35	3.37	3.04 × 10^−04^	5.31
	LysoPC(O-18:0)	3.37	5.08 × 10^−04^	1.66	4.57	5.32 × 10^−11^	2.21
	Oleic acid	1.41	4.01 × 10^−04^	0.74	1.86	3.45 × 10^−04^	0.75
	Arachidonic Acid (peroxide free)	1.25	4.31 × 10^−04^	0.78	3.66	1.96 × 10^−08^	0.41
	LysoPC(18:1(9Z))	2.90	8.94 × 10^−06^	0.48	1.92	1.24 × 10^−04^	0.63
	LysoPE(16:0/0:0)	3.53	1.59 × 10^−08^	1.59	6.35	1.11 × 10^−04^	2.00
	PE(16:0/18:1(9Z))	1.22	2.94 × 10^−07^	2.67	2.34	1.11 × 10^−09^	3.97
	PC(16:0/16:0)	4.22	1.30 × 10^−10^	4.99	4.18	2.97 × 10^−10^	3.98
	PS(18:0/18:1(9Z))	1.02	2.56 × 10^−07^	2.43	2.14	2.18 × 10^−08^	3.83
	PC(18:1(9Z)/18:1(9Z))	11.58	2.25 × 10^−08^	4.08	6.54	5.93 × 10^−07^	6.44
	SOPC	3.69	4.05 × 10^−09^	3.37	3.01	4.32 × 10^−09^	3.45
Energy	ADP	4.79	4.17 × 10^−06^	1.94	3.10	6.91 × 10^−07^	1.63
	GDP	1.41	1.24 × 10^−05^	0.72	1.41	1.24 × 10^−05^	0.72
	GTP	2.21	4.37 × 10^−03^	2.03	2.04	5.37 × 10^−04^	3.14
Nucleic acid	2′-O-methylcytidine	2.70	2.18 × 10^−10^	0.32	1.75	2.30 × 10^−07^	0.51
	CTP	1.50	8.34 × 10^−05^	1.56	2.08	3.40 × 10^−06^	1.65
	UTP	2.08	6.54 × 10^−04^	2.86	2.30	5.85 × 10^−04^	5.88
	dGTP	10.36	1.05 × 10^−05^	2.57	12.58	6.78 × 10^−08^	6.97
	Uridine	1.11	6.61 × 10^−07^	0.23	2.19	4.02 × 10^−09^	0.18
	Inosine	1.72	3.86 × 10^−09^	0.24	5.84	1.41 × 10^−07^	0.27
	Thymidine	2.05	2.01 × 10^−12^	0.12	4.59	3.33 × 10^−12^	0.13
	Adenosine 3′-monophosphate	2.50	3.86 × 10^−08^	1.79	2.33	2.24 × 10^−07^	3.34
	UDP	1.49	8.75 × 10^−04^	0.44	1.28	7.28 × 10^−05^	0.63
	UDP-D-Galactose	4.91	2.72 × 10^−06^	0.33	1.62	2.33 × 10^−06^	0.62

*VIP* Variable Importance for the Project, *FC* fold change, *NAAG* N-Acetylaspartylglutamate, *SOPC* 1-Stearoyl-2-oleoyl-sn-glycerol 3-phosphocholine, *ADP* adenosine 5′-diphosphate, *GDP* Guanosine 5′-diphosphate, *GTP* Guanosine 5′-triphosphate, *CTP* Cytidine triphosphate, *UTP* Uridine 5′-triphosphate, *dGTP* Deoxyguanosine triphosphate, *UDP* Uridine 5′-diphosphate

**Table 5:** Summary of differential metabolites between OSC and OSC-IC50

	Metabolite	143B			MG63		
		VIP	P value	FC	VIP	*P* value	FC
Amino acid	Lysyl-Glycine	2.59	1.30 × 10^−07^	0.05	2.30	5.17 × 10^−09^	0.30
	Diethanolamine	1.02	7.65 × 10^−07^	0.25	3.80	5.46 × 10^−05^	0.05
	Prolyl-Valine	2.02	1.56 × 10^−09^	0.06	1.63	6.52 × 10^−08^	0.34
	Alanyl-Isoleucine	1.44	5.13 × 10^−11^	0.05	1.24	1.38 × 10^−09^	0.27
	l-Proline	1.21	3.23 × 10^−06^	0.07	1.41	8.93 × 10^−05^	0.20
	L-prolyl-l-phenylalanine	1.85	2.95 × 10^−09^	0.12	1.53	4.61 × 10^−06^	0.52
	Phenylalanyl-Alanine	7.18	4.58 × 10^−13^	0.04	6.47	3.25 × 10^−11^	0.25
	Leucyl-Arginine	1.09	1.98 × 10^−09^	0.1	1.56	7.75 × 10^−09^	0.16
	Isoleucyl-Tyrosine	2.71	6.79 × 10^−11^	0.09	2.97	3.73 × 10^−12^	0.19
	Phenylalanyl-Glutamate	2.28	7.34 × 10^−10^	0.1	1.79	2.04 × 10^−11^	0.25
	S-Methyl-5′-thioadenosine	1.99	3.52 × 10^−06^	1.29	1.19	8.85 × 10^−07^	1.37
	Ornithine	1.07	2.35 × 10^−09^	0.07	1.42	1.60 × 10^−09^	0.10
	Valyl-Tryptophan	1.85	6.98 × 10^−07^	0.05	2.62	3.05 × 10^−12^	0.06
	Alpha-N-Phenylacetyl-l-glutamine	1.37	1.74 × 10^−09^	0.05	1.62	1.24 × 10^−13^	0.08
	Tyrosyl-Serine	1.56	8.97 × 10^−10^	0.08	1.35	1.11 × 10^−12^	0.20
	Phenylalanylphenylalanine	2.62	1.05 × 10^−4^	0.14	3.69	1.03 × 10^−08^	0.17
	Isoleucyl-Tryptophan	1.78	5.36 × 10^−4^	0.12	2.72	1.02 × 10^−07^	0.10
	Phenylalanyl-Tryptophan	1.59	5.23 × 10^−13^	0.06	1.24	1.35 × 10^−05^	0.39
	L-Targinine	1.70	4.56 × 10^−06^	0.04	1.43	1.90 × 10^−06^	0.05
	Methionyl-Valine	1.14	2.71 × 10^−05^	0.12	1.03	6.06 × 10^−05^	0.18
	Valyl-Methionine	1.72	1.38 × 10^−10^	0.03	1.84	1.56 × 10^−09^	0.04
	Lysyl-Threonine	1.22	1.35 × 10^−07^	0.09	1.07	4.67 × 10-08	0.16
	Leucyl-Glutamine	1.09	5.00 × 10^−07^	0.1	1.99	1.60 × 10^−08^	0.02
	l-Leucine	1.33	4.13 × 10^−05^	3.28	2.05	3.10 × 10^−07^	3.22
	Pantothenate	1.56	1.64 × 10^−05^	0.67	5.04	7.40 × 10^−11^	0.26
	Glutathione disulfide	1.41	5.28 × 10^−05^	1.23	2.37	4.21 × 10^−13^	15.32
	Harman	7.91	8.60 × 10^−12^	2.44	7.02	5.15 × 10^−14^	4.09
	7,8-Dihydrobiopterin	1.06	5.80 × 10^−4^	0.26	2.31	7.12 × 10^−12^	0.08
Fatty acid	Erucic acid	1.55	3.44 × 10^−07^	2	1.73	2.94 × 10^−11^	6.93
	Erucamide	13.40	1.33 × 10^−07^	2.25	14.23	4.21 × 10^−10^	9.40
	Phytanic acid	1.33	1.08 × 10^−08^	2.87	1.10	2.14 × 10^−06^	5.35
	Capric acid	1.74	4.95 × 10^−07^	1.36	1.69	1.35 × 10^−09^	1.78
	Undecanoic Acid	1.61	3.49 × 10^−10^	2.44	1.10	1.04 × 10^−10^	2.75
	L-Palmitoylcarnitine	13.90	2.50 × 10^−12^	0.29	9.14	2.22 × 10^−09^	0.70
	7-Ketocholesterol	1.01	4.16 × 10^−06^	1.51	1.46	6.46 × 10^−11^	5.45
	LysoPE(16:0/0:0)	3.85	4.89 × 10^−13^	0.31	4.14	4.15 × 10^−13^	0.19
	25-Hydroxycholesterol	2.77	1.90 × 10^−12^	6.03	1.92	8.11 × 10^−10^	9.34
	Undecanedioic acid	1.21	4.85 × 10^−07^	0.25	1.24	7.43 × 10^−11^	0.27
	Dihydrotachysterol	2.09	6.82 × 10^−10^	0.34	1.92	3.47 × 10^−08^	0.56
	LysoPC(18:0)	5.81	8.00 × 10^−09^	0.74	6.03	1.86 × 10^−10^	0.60
	LysoPC(16:0)	12.15	8.81 × 10^−12^	0.51	13.62	2.12 × 10^−13^	0.26
	LysoPE(20:3(8Z,11Z,14Z)/0:0)	1.37	3.84 × 10^−06^	0.57	1.09	1.89 × 10^−10^	0.58
	LysoPC(O-18:0)	3.87	2.12 × 10^−12^	0.41	1.55	1.88 × 10^−06^	0.84
	LysoPC(18:1(9Z))	1.20	6.82 × 10^−3^	1.67	2.02	4.62 × 10^−05^	2.67
	PC(16:0/16:0)	2.25	1.46 × 10^−4^	1.29	3.16	1.49 × 10^−10^	7.42
	PC(18:1(9Z)/18:1(9Z))	8.55	3.54 × 10^−07^	1.96	8.54	3.17 × 10^−13^	26.38
	SOPC	2.87	3.36 × 10^−2^	1.66	4.28	8.73 × 10^−15^	13.75
	Stearic acid	2.96	1.77 × 10^−07^	1.5	3.58	1.32 × 10^−11^	2.66
	LysoPE(16:0/0:0)	4.73	2.19 × 10^−10^	0.41	4.67	3.70 × 10^−13^	0.37
	UDP-d-Glucose	4.06	1.57 × 10^−05^	0.18	1.76	8.36 × 10^−06^	0.46
	Pentadecanoic Acid	2.02	2.90 × 10^−4^	5.05	1.11	3.11 × 10^−2^	4.57
	Geranyl diphosphate	1.86	2.04 × 10^−06^	1.33	2.49	3.13 × 10^−12^	2.81
Energy	DL-lactate	3.03	4.13 × 10^−08^	0.46	2.36	5.01 × 10^−08^	0.51
	Acetoin	2.08	1.25 × 10^−06^	0.55	1.22	7.97 × 10^−05^	0.71
Drug	Naproxen	1.13	1.78 × 10^−09^	0.12	1.01	1.90 × 10^−08^	0.20
	Promazine	1.53	2.22 × 10^−10^	0.06	2.19	6.63 × 10^−12^	0.14
	Desipramine	1.85	3.01 × 10^−3^	0.18	2.62	2.92 × 10^−4^	0.24
Nucleic acid	Thymidine	1.20	1.34 × 10^−09^	0.07	1.10	2.59 × 10^−09^	0.37

*VIP* Variable Importance for the Project, *FC* fold change, *SOPC* 1-Stearoyl-2-oleoyl-sn-glycerol 3-phosphocholine, *UDP-d-Glucose* Uridine diphosphate glucose

**Table 6:** Summary of differential metabolites between OS-IC50 and OSC-IC50

	Metabolite	143B			MG63		
		VIP	P value	FC	VIP	P value	FC
Amino acids	Prolyl-Threonine	2.23	9.27 × 10^−13^	0.07	1.56	1.07 × 10^−09^	0.33
	Histidinyl-Methionine	1.54	9.25 × 10^−07^	3.23	4.10	2.82 × 10^−08^	8.27
	S-Methyl-5′-thioadenosine	2.31	3.54 × 10^−02^	1.60	2.07	6.89 × 10^−08^	1.47
	Isoleucyl-Valine	1.77	2.33 × 10^−04^	1.31	3.42	3.10 × 10^−10^	13.49
	Glutathione disulfide	1.98	2.59 × 10^−04^	1.60	3.06	1.58 × 10^−09^	11.05
	l-Phenylalanine	3.80	8.28 × 10^−05^	3.54	1.54	7.72 × 10^−05^	1.44
	l-Tryptophan	4.17	1.10 × 10^−12^	3.70	1.61	1.67 × 10^−06^	1.43
Fatty acids	PG(16:0/18:1(9Z))	2.14	1.32 × 10^−03^	0.32	1.59	1.70 × 10^−07^	0.36
	N-Oleoylethanolamine	2.36	3.55 × 10^−06^	2.01	1.70	8.55 × 10^−06^	1.66
	Stearoylcarnitine	7.76	2.80 × 10^−13^	0.11	5.18	1.67 × 10^−04^	0.81
	LysoPE(16:0/0:0)	2.27	1.40 × 10^−04^	0.76	4.66	1.03 × 10^−09^	0.55
	LysoPE(20:3(8Z,11Z,14Z)/0:0)	1.49	2.08 × 10^−04^	0.52	1.58	8.2 × 10^−08^	0.61
	LysoPC(18:1(9Z))	4.11	2.08 × 10^−07^	6.03	2.05	2.01 × 10^−04^	1.78
	Phosphorylcholine	1.17	6.13 × 10^−05^	0.59	1.22	5.24 × 10^−06^	0.41
	Arachidonic Acid (peroxide free)	1.25	1.16 × 10^−03^	1.23	2.38	2.08 × 10^−06^	1.61
	LysoPE(16:0/0:0)	4.17	1.33 × 10^−08^	0.60	2.64	3.96 × 10^−02^	0.73
	Cholesterol sulfate	1.92	4.56 × 10^−05^	4.87	1.32	3.81 × 10^−06^	8.33
	Geranyl diphosphate	3.51	1.67 × 10^−13^	0.03	1.95	1.72 × 10^−09^	0.08
Nucleic acid	Inosine	1.86	2.57 × 10^−09^	3.44	5.39	1.50 × 10^−08^	12.82
	Uridine	1.14	2.49 × 10^−06^	2.91	1.83	3.42 × 10^−09^	6.46
	Thymidine	2.05	2.68 × 10^−11^	3.36	3.75	4.10 × 10^−12^	6.95
	2′-O-methylcytidine	2.51	8.08 × 10^−07^	0.68	4.33	1.19 × 10^−09^	0.25
	dGTP	4.71	2.72 × 10^−02^	0.72	4.00	1.28 × 10^−04^	0.50

*VIP* Variable Importance for the Project, *FC* fold change, *dGTP* Deoxyguanosine triphosphate

## Discussion

In the past several decades, emerging evidence indicates that CSC is considered to have the ability to retain stem cell-like properties through self-renewal and differentiation and be responsible for tumor initiation, propagation, recurrence and resistance to therapy [14, 39]. Moreover, CSC has now been suggested to bear a distinct metabolic phenotype and targeting CSC metabolism may provide a selective advantage to eventually take over and drive relapse of cancers [39, 40]. Increasing evidence has indicated the existence of CSCs in OS [29, 41] and yet, the mechanism of these CSCs in the progression of OS remains vague. Recent studies using metabolomics methodologies have revealed possible biomarkers for diagnosis and prognosis of sarcomas [42], mechanisms of metastasis [24] and response to chemotherapeutic agents [25, 43] in OS. However, little is known about the metabolism of OSCs and their response to the chemotherapeutic agent(s).

In the present study, though several methodologies were employed to isolate stem cells from parental cancer cells [13, 29, 41], sphere-forming assay was used to efficiently isolate OSCs and the isolated cells showed enhanced expression of SC genes and tumorigenic potential of OSCs in vitro and in vivo, as well as enhanced resistance to MTX. After isolation and identification of OSCs, the untargeted LC–MS/MS analysis was employed to initially investigate the metabolic feature of OSCs and the feature of their response to MTX.

The metabolomics methodology has been used to investigate some characteristics of CSCs. Previously, in the study of glioblastoma [44], magnetic resonance spectroscopy (MRS) was employed to identify CSCs and discover new diagnostic or prognostic biomarkers. Brandi et al. [18] employed LC–MS/MS analysis to prove that specific characteristics of pancreatic CSCs were identified with differentially expressed intracellular proteins and corresponding pathways. While in a recent study on thyroid carcinoma [45], cancer stem-like cells showed significant differences in Krebs cycle intermediates, amino acids, cholesterol, and fatty acids content, compared to non-cancer stem-like cells. However, they did not report the signaling pathways involved in characterization of CSCs. In our present study, the metabolomics results showed that differential metabolites and perturbed pathways between parental OS cells and their SCs were mainly involved in metabolisms of fatty acid, amino acid and nucleic acid. Dissimilarities was observed in previous study, compared with our results, which might be the uniformity of definition and isolation techniques for CSCs [46]. In addition, the origins of CSCs within a cancer have not been clarified, oncogenic transformation from progenitor cells or normal tissue stem cells might be one of possible mechanisms [14], which might be accompanied with abnormal nucleic acid metabolism, such as DNA methylation [47] or RNA expression [48].

Moreover, one of the most important issues about cancer therapy is that increasing evidence has shown chemotherapeutic resistance in cancers, which is considered partially due to the existence of CSCs [14]. Studies have been carried out to investigate underlying mechanisms of chemotherapeutic resistance in CSC. However, the conclusion remains vague and little is known about the metabolic characteristics of OSC responding to chemotherapeutic agents. Lamego et al. [25], found drug-specific metabolic features in MG63 cells, by comparing metabolomics of OS cells responding to doxorubicin (DOX), methotrexate (MTX) and cisplatin (cDDP). In addition, MTX-treated cells showed no lipids increase and different phospholipid features, which suggests that MTX induces decreased membrane synthesis, while no membrane disruption or de novo lipid synthesis seem to occur. Furthermore, Lamego and collegues [43] assessed the impact of the potential palladium drug, Pd2Spermine Chelate, on cell metabolism and the results illustrated the ability of NMR metabolomics to measure cellular responses to different drugs. In the present study, the results of untargeted LC–MS/MS analysis showed that after MTX treatment at an IC50 concentration, OSCs showed specific metabolic feature with differential metabolites and perturbed pathways, involved in amino acid, fatty acid, nucleic acid and energy metabolisms, compared with their parental OS cells. Besides, Rap1 signaling pathway and Ras signaling pathway were identified both in parental OS cells and their SCs. Rap1 and Ras signaling was suggested to involve in cancer migration, invasion, proliferation and gene expression and activation [37, 38]. Previous studies showed that Rap1 and Ras signaling pathways might be involved in OS proliferation, migration, invasion and metastasis [49–51]. Moreover, Rap1 signaling pathway is found to be deregulated in cancer stem-like cancer cells [52] and Ras signaling pathway was involved in cancer stem cell expansion, suggesting both signaling pathways might play important roles in OSC progression. What’s more, Ras signaling was suggested a target in treating cancers [53]. However, the mechanism for MTX modulating Ras signaling remains vague. Previously, studies showed Ras methylation in colon cancer cells was decreased after methotrexate treatment of colon cancer cells and this hypomethylation was accompanied by a mislocalization of Ras to the cytosol [54]. Besides, Ras activation was suggested responsible for the subsequent perturbation of the MTX-mediated G1 cell cycle restriction [55]. As for Rab1 signaling pathway, studies showed that Rab1A might be an mTORC1 activator and a potential target in treating cancer progression [56]. Further investigations were encouraged to clarify the mechanism of Rab1 signaling and Ras signaling in chemotherapeutic treatment for cancer.

## Conclusions

In conclusion, after isolating and identifying OSCs, metabolomics methodology was employed to measure the differential metabolites and perturbed signaling pathways between parental OS and their SCs, and cell response to MTX. The results suggested LC–MS/MS analysis a sufficient methodology to investigate metabolic features of OS cells and OSCs. Moreover, the metabolomics features of OSCs response to MTX might reveal a further understanding of chemotherapeutic resistance in OS.

## Supplementary information

**Additional file 1.** Standard procedures for isolation and identification of OS stem cells.

## Data Availability

The datasets generated and analyzed during the current study are included in the published article and available from the corresponding author on reasonable request.
