# Supplementary material for: Untargeted LC–MS/MS analysis reveals metabolomics feature of osteosarcoma stem cell response to methotrexate
Source: Cancer Cell Int. 2020 Jun 24;20:269. doi: 10.1186/s12935-020-01356-y (PMC7313215; doi:10.1186/s12935-020-01356-y)
Supplement: Supplementary file 1 — Additional file 1. Standard procedures for isolation and identification of OS stem cells. [file 12935_2020_1356_MOESM1_ESM.docx]

**Additional file 1. Standard procedures for** **isolation and identification of OS stem cells**

***Sphere‑forming assay.***

The human OS 143B and MG63 cell lines were (kindly gifted by Professor Zhengdong Cai, First Hospital of Shanghai, Shanghai, China) used to isolate cancer stem cells (CSCs), which were enriched in Dulbecco's modified Eagle's medium (DMEM) and RPMI 1640 (Sigma-Aldrich, St. Louis, MO, USA), supplemented with 10% fetal bovine serum (Gibco, Grand Island, NY, USA) at 37°C in a humidified incubator with 95% air and 5% CO2. Sphere-forming assay was performed as described previously[7,25]. Briefly, after reaching confluence, cells were harvested and plated at a density of 60,000 cells/well on 6-well poly-HEMA-coated plates (Sigma-Aldrich) in serum-free DMEM and RPMI 1640 medium with 1% of methylcellulose (Sigma-Aldrich) supplemented with penicillin/streptomycin (Gibco), 20 nM progesterone (Sigma-Aldrich), 100μM putrescine (Sigma-Aldrich), 1% insulin-transferrin-selenium supplement (Gibco), 10 ng/ml basic fibroblast growth factor (bFGF, Peprotech EC, London, UK) and 10 ng/ml human recombinant epidermal growth factor (hEGF, Sigma-Aldrich). After 7 days of culturing with fresh aliquots of growth factors added twice a week, the formed cellular spheres (sarcospheres) were collected and re-seeded again in stressful growth conditions to form secondary spheres, which was repeated at least 3 times. The total number of spherical colonies was quantitated and sphere-forming efficiency was calculated at each passage. A third generation sphere culture was transferred to grow in monolayer in culture media supplemented with 10% FBS without growth factors. This sphere-derived monolayer culture was expanded and used in subsequent studies, which were referred as 143B SCs (143B-SCs) and MG-63 SCs (MG63-SCs), respectively.

***Immunofluorescent staining***

Immunofluorescent staining was performed to evaluate the expression of OCT-4 and CD133 as described previously [23]. Briefly, parental OS cells and their SCs were pre-treated with DATS in 24-well culture plates. All samples of cells were washed three times in PBS and then fixed in PBS containing 4% formaldehyde for 30 min at room temperature. The cells were then washed twice in PBS and incubated for 30 min in PBS containing 0.1% Triton X-100. Next, the cells were blocked with 200 µl 10% normal goat serum diluted with PBS and incubated for 30 min at room temperature. Thereafter, the cells were incubated with 100 µl primary OCT-4 antibody (1:1000) or CD133 antibody (1: 1000) per well in a moist chamber at 4°C overnight. After washing three times with PBS, the cells were incubated with DAPI for 5 min at room temperature.

The cells were then analyzed using a Leica DM4000B microscope (Leica Microsystems GmbH, Wetzlar, Germany). Images were captured and expression levels determined using the Image-Pro Plus image analysis system 7.0 (Media Cybernetics, Inc., Rockville, MD, USA).

***RNA extraction and qRT‑PCR***

Quantitative real-time polymerase chain reaction (qRT-PCR) was performed, as described previously [29], to evaluate the expression of CD117 at a transcriptional level. Total RNA was isolated from OS cells and their SCs, using TRIzol reagent (Invitrogen) according to the manufacturer's instruction. The following PCR conditions were used: 45 cycles of 30 sec at 95℃, 30 sec at 58℃, and 60 sec at 72℃. Primer sequences of CD117 and GAPDH were synthesized and purchased from Beijing Genomics Institute (BGI, China), which were F: TTCCCCAAACCTGAACACCA, R: AACAGGGTGTGGGGATGGATT and F: ACAACTTTGGTATCGTGGAAGG, R: GCCATCACGCCACAGTTTC, respectively. Relative fold change in mRNA expression compared to control was calculated using the comparative Ct method.

***Immunoprecipitation and Westernblot***

Immunoprecipitation was performed as previously described [29]. Briefly, after proteins were extracted from OS cells and their SCs, the total protein concentration was determined. The supernatants were collected and primary antibodies (5 µg/ml) of ALDH1 (Proteintech), Sox2 (Proteintech) and GAPDH (Abbkine) pre-conjugated with magnetic beads (50 µl; Invitrogen) were added to the supernatant and incubated with gentle agitation, respectively. Proteins bound to the beads were eluted and loaded for sodium dodecyl sulfate-polyacrylamide gel electrophoresis and thereafter transferred to polyvinylidene fluoride membranes. The membranes were blocked and incubated with the primary antibodies overnight at 4˚C. Then, the membranes were incubated with secondary antibodies at room temperature for 1 h, and the signal was detected using a Tenon GIS gel image system (Shanghai, China)

***Matrigel® migration/invasion assay.***

Matrigel® migration/invasion assay was performed as previously described [29] with minor modification. Briefly, the concentration of parental OS cells and their SCs was adjusted to 10^5^/ml at room temperature, then a total of 10^4^ cells were transferred and incubated in the upper chamber for 12 h with or without Matrigel® plated on the bottom of the chamber for migration or invasion assay. Next, the culture media were removed and the cells were fixed with 4% formaldehyde for 15 min at room temperature. After washed with PBS for 3 times, the cells in the lower chamber were stained with crystal violet for 1 h at room temperature. When the chambers were dried out, the cells in the upper chamber were removed carefully. The migrated and invasive cancer cells were then visualized and counted from five different visual fields at x100 magnification under an inverted microscope to compare migration/invasion rate. The assay was performed in triplicate. The cell migration/invasion rate was calculated as: (No. of cells in lower chamber/ No. of cells incubated in upper chamber) x 1000.

***CCK-8 assay***

The proliferation activity of parental and their SC cells *in vitro* was measured by CCK-8 assay (Transgen, Beijing), according to the manufacturer's instructions. Cells were plated in 24-well plates (5x10^4^cells/well) and cultured at 37˚C. After 0h, 6h, 12h, 24h and 48h of incubation, 20 µL CCK-8 reagent was added to each well, and incubated for additional 4h. Absorbance of each well was determined at 450 nm. The absorbance obtained were proportional to the number of viable cells. The experiments were repeated trice to confirm the reproducibility.

***Orthotopic xenograft animal experiments.***

Orthotopic xenograft models were performed as previously described [20, 29]. Briefly, OS cells and their SCs were harvested and diluted in 100 µl serum-free medium and subcutaneously injected (1.5x10 ^5^ cells/injection) into the left flanks of BALB/c nude mice. Three mice were used in each group. At 3 weeks after injection, the mice were sacrificed by CO_2_ inhalation. Tumor volume (TV) was calculated using the following formula: TV (mm^3^) = (length x width^2^)/2.
